# Perinatal Epidemiology When Data Are Imperfect: Lessons From Studies on Maternal Mortality

**DOI:** 10.1111/ppe.70017

**Published:** 2025-04-01

**Authors:** Jennifer Zeitlin

**Affiliations:** ^1^ Centre for Research for Epidemiology and Statistics (CRESS‐UMR1153), obstetrical, Perinatal and Pediatric Lifecourse Epidemiology (OPPaLE) Université Paris Cité and Université Sorbonne Paris Nord, Inserm, INRAE Paris France

This issue of *Paediatric and Perinatal Epidemiology* includes three commentaries on the article by Joseph and colleagues that appeared in the April 2024 issue of the American Journal of Obstetrics and Gynecology entitled ‘Maternal mortality in the United States: are the high and rising rates due to changes in obstetrical factors, maternal medical conditions or maternal mortality surveillance?’ [1]. This publication triggered a debate on how to monitor an essential indicator of population health when data are imperfect. To provide insight into these measurement issues for our readership, we commissioned three commentaries from researchers with in‐depth knowledge of maternal mortality measurement from their roles as chairs of Maternal Mortality Review Committees (MMRC) and National Confidential Enquiries and their contributions to scholarly research [2, 3, 4]. They were invited to comment on the publication and provide a broader vision for future research. The authors of the AJOG publication were also invited to respond to the commentaries [5]. We thank all the contributors for sharing their time and expertise. Note that Cande Ananth, Editor‐in‐Chief of *Paediatric and Perinatal Epidemiology* and a coauthor of the AJOG publication, was not involved in the editorial process of commissioning, management, and peer review of the commentaries, and the response from Joseph and colleagues.

At the heart of this debate is how to use and present imperfect data in epidemiological research and, more specifically, whether the vital statistics data used in the AJOG study can provide accurate information about the rates and temporal trends in maternal death. The debate about using data with methodological limitations for studies of maternal mortality is not new, as KS Joseph and colleagues remind us in their response to the commentaries. They cite the editorial by Richard Horton, editor of the Lancet, in response to calls to delay a publication on global rates and trends in maternal mortality in 2010 where he asks ‘Is the global health community unable to accommodate diverse voices and sources of evidence? Is it unable to create constructive ways to bring scientists and policymakers together to reach agreement about the meaning of new research findings?’ [6] In their response to Horton, reviewers of the Lancet study agree with the need to publish new data, but contend that adequately addressing measurement issues should be paramount as ‘the responsibility of measurement scientists today is to go the extra mile and “translate” findings for an increasingly wide variety of end users or else to risk misinterpretation and confusion’ [7].

Imperfect data is not a problem unique to maternal mortality; however, the maternal mortality rate may be one of the most difficult indicators to measure well. The three commentaries describe this complexity in detail. As Elliot Main points out, unlike other indicators which can be produced with one data source ‘accurate assessment of maternal deaths requires multiple data sources and a series of judgements’. False negatives are common in death certificates because mention of pregnancy is often omitted, whereas false positives are frequent, especially those linked to the pregnancy check box. Triangulating data from multiple sources, including linking deaths of women of childbearing age to births, is important to reduce false positives and false negatives, but is not enough to provide reliable information on the causes and circumstances of maternal deaths. Marian Knight and Catherine Deneux‐Tharaux provide a set of enhanced ascertainment methods needed to achieve this objective. These methods have been implemented in national confidential enquiries into maternal deaths in France and the UK and in MMRC in some US states. Other challenges, such as changes in coding rules from ICD‐9 to ICD‐10, complicate the assessment of trends in causes of death, as illustrated by Main in his commentary.

The commentaries also underscore the challenge of presenting data on maternal mortality to a non‐specialist and lay public. As an epidemiologist well versed in indicator development, I found it daunting to understand the multiple definitions used for the maternal mortality rate, the varied data sources and their specific limits which yield rates for the US ranging from lows of 8 to highs of 30 per 100,000 live births (for others feeling similarly confused about the US situation, this article is helpful [8]).

How then, going forward, can we ‘bring scientists and policymakers together to reach agreement’ [6]? One suggestion is to produce guidelines for analysis and reporting of indicators, such as the maternal mortality rate, which are known to be unreliable and difficult to compare. Including stakeholders in defining these guidelines could ensure that research results are less vulnerable to misinterpretation. Relevant stakeholders include data coders and managers, researchers involved in data production, clinicians doing mortality reviews, policymakers, maternal health advocates, communities and patients. These guidelines could recommend strategies for presenting data in a way that communicates the uncertainty of the results, the need for data improvement and the value of data‐driven insights in deepening knowledge and informing future research. An example is probabilistic bias analysis. In their response, KS Joseph and colleagues provide a bias analysis showing that their conclusion of no increase in maternal mortality was robust to a variety of assumptions about under and over reporting of maternal deaths, although they call attention to the limits of underlying hypotheses. Corrected rates were 16.7 per 100,000 births in 1999–2002 and 16.1 per 100,000 live births in 2018–2021, substantially higher than the published estimates of 10.2 and 10.4 per 100,000 live births in these periods, respectively. This analysis makes it possible to acknowledge the uncertainty of the data, while providing information on the trend.

Leveraging MMRC reviews which use multiple data sources and can calculate false positive and false negative rates for sub‐groups by race and ethnicity or other socioeconomic characteristics could be used for more in‐depth probabilistic bias analysis, as suggested by Teresa Janevic, Eugene Leclerc and Elizabeth Howell in their commentary. Incorporating high‐quality local data into analyses of national trends could increase external validity and calls attention to valuable, resource‐intensive initiatives that often go unseen and may not be prioritised in public health spending.

Although there are varying stances in this debate, the AJOG article and commentaries also reveal areas of common ground. The first area of agreement is the existence of stark and persistent racial and ethnic disparities in maternal mortality which is highlighted in the AJOG article and the commentaries. As noted by Main ‘every version of maternal mortality measures shows disparities of at least 3‐fold for Black and Native American mothers’ in the United States. The consistency of this finding illustrates the challenge of achieving equity and the need for ‘centring equity in the maternal mortality surveillance debate’ [2]. The second area of agreement relates to chronic conditions, including mental health problems, which are increasing among women of childbearing age. All contributors acknowledge the increasing difficulties of determining the causal link between the death and the pregnancy when the cause is related to underlying medical conditions, as opposed to obstetric conditions, and as time passes since the delivery. All argue for broadening the scope of inquiry. Knight and Deneux‐Tharaux note that pregnancy may not cause a death, but could lead to inappropriately altered management of chronic conditions or other diseases that raise the risk of death, such as foregone cancer screening. Janevic and colleagues note that: ‘from the vantage point of potential interventions, the measure of pregnancy‐associated death may be more relevant and comprehensive… public health interventions have an opportunity to prevent deaths in the postpartum period regardless of classification. This point is especially relevant given that deaths from suicide and substance use disorders are rising’ [2]. As pregnant women and new mothers are generally in better health than non‐pregnant women, this more comprehensive definition provides an opportunity for prevention in this lower risk population and a springboard for developing strategies targeting all women of childbearing age.

These insights on maternal mortality measurement are relevant for other perinatal indicators. For instance, stillbirth rates computed using routine birth data are hampered [9] by inconsistent indicator definitions, unreliable data sources and evolving practices in survival‐focused care for extremely preterm births. As suggested above for maternal mortality, these limitations could be addressed by developing stakeholder‐informed reporting guidelines, supplementing routine data with comprehensive local studies, and moving beyond individual metric‐related debates to emphasise the broader issues—such as ethnic, racial and socioeconomic inequalities and holistic views of pregnancy loss.

Although more data are available today for research, they remain inadequate, even in high‐income countries [10]. The challenge for epidemiologists is to champion high‐quality data by educating policymakers and the media about its limits, whereas simultaneously using data to generate and share evidence that illuminates the current crises in maternal and child health and drives actions for better, more equitable maternal outcomes.

## Author Contributions

J.Z. is the sole contributor to this editorial.

## Conflicts of Interest

The author declares no conflicts of interest.

## Data Availability

The author has nothing to report.
